# Complete Genome Sequences of Six Lactobacillus iners Strains Isolated from the Human Vagina

**DOI:** 10.1128/MRA.00234-20

**Published:** 2020-05-14

**Authors:** Michael T. France, Lindsay Rutt, Shilpa Narina, Sarah Arbaugh, Elias McComb, Mike S. Humphrys, Bing Ma, Matthew R. Hayward, Elizabeth K. Costello, David A. Relman, Douglas S. Kwon, Jacques Ravel

**Affiliations:** a Institute for Genome Sciences, University of Maryland School of Medicine, Baltimore, Maryland, USA; b Department of Microbiology and Immunology, University of Maryland School of Medicine, Baltimore, Maryland, USA; c Ragon Institute of MGH, MIT, and Harvard, Cambridge, Massachusetts, USA; d Division of Infectious Diseases & Geographic Medicine, Department of Medicine, Stanford University School of Medicine, Stanford, California, USA; e Department of Microbiology & Immunology, Stanford University School of Medicine, Stanford, California, USA; f Infectious Diseases Section, Veterans Affairs Palo Alto Health Care System, Palo Alto, California, USA; g Division of Infectious Diseases, Massachusetts General Hospital, Boston, Massachusetts, USA; Loyola University Chicago

## Abstract

Lactobacillus iners is a common member of the human vaginal microbiota, with a genome size smaller than that of other lactobacilli. Here, we report the complete genome sequences of six *L. iners* strains isolated from different vaginal swab specimens. Three strains were found to harbor ∼100-kbp plasmids, which were not known previously.

## ANNOUNCEMENT

Lactobacillus iners is a Gram-positive, facultative anaerobic bacterium that is a member of the human vaginal microbiota (1, 2). Surveys have demonstrated that a substantial portion of reproductive-age women have a vaginal microbiota dominated by *L. iners* (3, 4). The species’ relationship to vaginal health is somewhat complicated (5). While it is capable of lowering vaginal pH via the production of lactic acid, a hallmark of vaginal health (6), it is also frequently found coresident with species that have been associated with bacterial vaginosis, a common vaginal condition associated with adverse health outcomes (7–9). The species has also been shown to provide suboptimal protection against sexually transmitted infections, such as those caused by chlamydia (10, 11). Here, we report the complete genome sequences of six *L. iners* strains.

The six *L. iners* strains were isolated from archived and deidentified midvaginal swab specimens collected from either African American (*n* = 3) or Caucasian women (*n* = 3). The swab specimens were originally collected after obtaining informed consent from all participants, who also provided consent for storage of the samples and their use in future research studies related to women's health. The original studies were approved by the University of Maryland School of Medicine institutional review board. The swab specimens were resuspended in 1 ml of brucella broth supplemented with hemin and vitamin K, and then 25 μl of the suspension was plated onto human blood bilayer agar with Tween 80. After 48 to 72 hours of aerobic or anaerobic (5%:10%:85% H_2_/CO_2_/N_2_ gas mixture) incubation at 37°C, the strains were isolated. Large genomic DNA fragments were extracted using the MasterPure complete DNA purification kit (Lucigen, Middleton, WI, USA). Sequencing libraries were prepared using the SMRTbell express template prep kit 2.0 (Pacific Biosciences of California, Menlo Park, CA, USA), size selected on a BluePippin instrument (Sage Science, Beverly, MA, USA), and sequenced on a PacBio Sequel II instrument (Pacific Biosciences) with a single-molecule real-time (SMRT) cell 8M.

An average of 310,000 reads were obtained for each strain (median read length, 10.1 kbp), providing an average genome coverage of 2,258× (range, 1,203× to 3,682×). Long read assembly was performed using the Canu assembler v1.8 on the raw PacBio reads with a target genome size of 1.3 Mbp and a minimum read length of 1 kbp (12). For three of the strains, a single large contig was produced, circularized using Simple-Circularise (https://github.com/Kzra/Simple-Circularise; v1 default settings), and then oriented using Circlator v1.5.5 with default settings (13), such that the origin of replication was at the start. The remaining three strains had an additional contig encoding an ∼100-kbp plasmid, which was also circularized.

**FIG 1:**
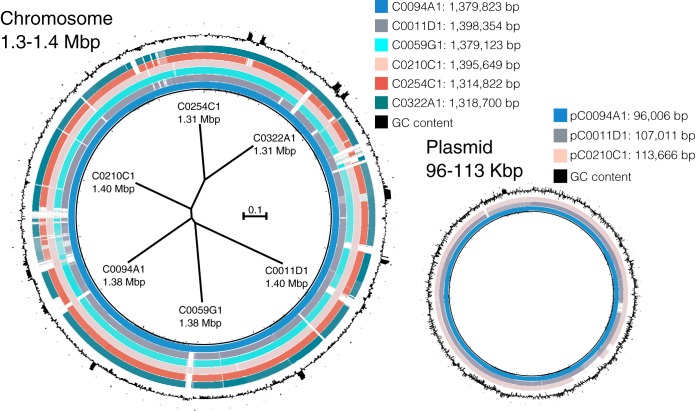
Circular whole-genome alignment of the six Lactobacillus iners chromosomes and the three identified plasmid sequences. Inside the chromosomal alignment is an unrooted phylogram derived from single nucleotide polymorphisms (SNPs) identified in the whole-genome alignment that displays the phylogenetic relationships and genome sizes of the six strains. Alignment figures were constructed using BRIG v0.95 (16) and the phylogram using CSI phylogeny v1.4 with default settings (17).

The average genome size of the six *L. iners* was 1.36 Mbp and ranged from 1.32 Mbp to 1.40 Mbp, with an average GC content of 33.3% (Fig. 1). Despite being isolated from six different subjects, the *L. iners* genome sequences were fairly similar, with an average nucleotide identify (ANI) of 98.7% and overlap of 91.6%, as estimated by pyani v0.2.10 with the -ANIm setting (14). The three plasmid sequences were also largely similar, with an ANI of 98.1% and overlap of 85.1%. Gene prediction and annotation were performed using Prokka v1.12 with default settings (15). The average number of coding sequences per genome was 1,254 and ranged from 1,203 to 1,331. Five of the six *L. iners* genomes were found to encode 6 rRNA operons, while the genome of strain C0254C1 encoded one fewer. All of the genomes encoded 71 tRNA genes, except the genome of strain C0059G1, which encoded 73. The plasmid was found to encode between 97 and 100 genes, including several related to conjugal transfer.

### Data availability.

The six genome sequences and three plasmid sequences have been deposited in the NCBI GenBank with accession numbers CP049223 to CP049231. Raw sequencing reads were submitted to the NCBI Sequence Read Archive under the BioProject PRJNA608123.
